# Synthesis and Biological Evaluation of Resveratrol Methoxy Derivatives

**DOI:** 10.3390/molecules28145547

**Published:** 2023-07-20

**Authors:** Elizabeth Fragopoulou, Katerina Gkotsi, Filio Petsini, Katerina Gioti, Amalia D. Kalampaliki, George Lambrinidis, Ioannis K. Kostakis, Roxane Tenta

**Affiliations:** 1 Department of Nutrition and Dietetics, Harokopio University, 70 Eleftheriou Venizelou Avenue Kallithea, 17676 Athens, Greece; gkotsi@hua.gr (K.G.); fpetsini@hua.gr (F.P.); catherine_geo@yahoo.com (K.G.); rtenta@hua.gr (R.T.); 2 Department of Pharmacy, Division of Pharmaceutical Chemistry, National and Kapodistrian University of Athens, 15771 Athens, Greece; akalampa@pharm.uoa.gr (A.D.K.); lamprinidis@pharm.uoa.gr (G.L.); ikkostakis@pharm.uoa.gr (I.K.K.)

**Keywords:** platelets, cancer, platelet activating factor, thrombin, ADP, resveratrol, methoxy

## Abstract

Resveratrol, a naturally occurring stilbene, exhibits numerous beneficial health effects. Various studies have demonstrated its diverse biological actions, including anti-oxidant, anti-inflammatory, and anti-platelet properties, thereby supporting its potential for cardio protection, neuroprotection, and anti-cancer activity. However, a significant limitation of resveratrol is its weak bioavailability. To overcome this challenge, multiple research groups have investigated the synthesis of new resveratrol derivatives to enhance bioavailability and pharmacological activities. Nevertheless, there are limited data on the effects of resveratrol derivatives on platelet function. Therefore, the objective of this study was to synthesize resveratrol methoxy derivatives and evaluate their anti-platelet and anti-proliferative activity. Platelet-rich plasma (PRP) obtained from healthy volunteers was utilized to assess the derivatives’ ability to inhibit platelet aggregation induced by platelet activating factor (PAF), adenosine diphosphate (ADP), and thrombin receptor activating peptide (TRAP). Additionally, the derivatives’ anti-tumor activity was evaluated against the proliferation of PC-3 and HCT116 cells. The results revealed that some methoxy derivatives of resveratrol exhibited comparable or even superior anti-platelet activity compared to the original compound. The most potent derivative was the 4′-methoxy derivative, which demonstrated approximately 2.5 orders of magnitude higher anti-platelet activity against TRAP-induced platelet aggregation, indicating its potential as an anti-platelet agent. Concerning in silico studies, the 4′-methyl group of 4′-methoxy derivative is oriented similarly to the fluorophenyl–pyridyl group of Vorapaxar, buried in a hydrophobic cavity. In terms of their anti-tumor activity, 3-MRESV exhibited the highest potency in PC-3 cells, while 3,4′-DMRESV and TMRESV showed the greatest efficacy in HCT116 cells. In conclusion, methoxy derivatives of resveratrol possess similar or improved anti-platelet and anti-cancer effects, thereby holding potential as bioactive compounds in various pathological conditions.

## 1. Introduction

Resveratrol is a naturally occurring stilbene with several beneficial health effects [1,2]. It is most commonly in grapes and wine but also in other plants such as polygonum cuspidatum, peanuts, and red fruits [2]. It exists in two diastereoisomeric forms found in nature: *trans*-form (E-1,2-diphenylethylene) and *cis*-form (Z-1,2-triphenylethylene), with the former being the bioactive arrangement. One hydroxyl group is present in one ring, in position 4′, and two hydroxyl groups are present in the other ring, in positions 3 and 5, respectively.

The study of its biological actions expanded after the introduction of the term “French Paradox” in 1992 by Renaud S and De Lorgeril [3]. Since then, several studies have shown that resveratrol exhibits a wide range of biological actions, including anti-oxidant, anti-inflammatory, and anti-platelet properties that support its cardioprotective, neuroprotective, and anti-cancer effects [4,5,6]. In this line, our previous study revealed that resveratrol possesses potent anti-oxidant activities since it could scavenge free radicals, inhibit LOX, and inhibits serum oxidation induced by Cu^2+^ and binds Fe^2+^ [7]. We have also shown that resveratrol has potent anti-platelet and anti-inflammatory activities since it inhibits platelet activating factor (PAF)-induced washed rabbit platelet aggregation [8] and PAF biosynthetic enzymes in unstimulated and IL-1-stimulated U937 monocytes cells [9,10] and possesses anti-cancer effects [11]. It should be noted that PAF is a potent lipid mediator of inflammation and platelet aggregation that participates in atherosclerosis, cancer, and neurodegeneration [12,13,14].

Although resveratrol has potent biological activities, its water solubility results in weak bioavailability [15]. To overcome this problem, several research groups have studied the synthesis of new resveratrol derivatives in order to enhance their bioavailability and pharmacological efficacy [16,17]. Recent reports indicate that resveratrol derivatives with methoxy, hydroxy, acetyl, or halogen groups could be potential agents against inflammation, cancer, neuron damage, and microbial or fungal infections [18,19]. Among them, methylation of aromatic hydroxyl groups consists of a promising structural modification. The O-methylation in the stilbene ring increases lipophilicity and has been shown to promote cell uptake, protect from degradation, and improve stability [20,21]. Also, methoxy derivatives revealed greater bioavailability and total plasma levels of the parent compound in rats [22,23].

Limited data exist regarding the effects of resveratrol derivatives on platelet function. Platelets are small discoid cytoplasmic fragments that come from megakaryocytes or lung cells. They do not have a nucleus, but they retain the ability for mRNA and protein synthesis [24]. Platelets are crucial in primary hemostasis and thrombus formation, but recent evidence suggests their involvement in diseases such as atherosclerosis, cancer, diabetes, and neurodegeneration [25]. We have previously shown that resveratrol acetylated derivatives could inhibit platelet aggregation against PAF [26]; however, there is only one recent study that found that the 3,5,2′,4′-tetramethoxy derivative of resveratrol inhibits thrombin-induced platelet aggregation [27].

Furthermore, the connection between inflammation and malignancy is now well-recognized. Platelets play significant roles in cancer progression by providing surface and granular contents for several interactions as well as behaving like immune cells. Anti-platelet agents may prevent cancer, decrease tumor growth and metastatic potential, and improve cancer patients’ survival [28,29]. 

Therefore, the present study aims to synthesize methoxy derivatives in the existing hydroxyl groups of resveratrol and to evaluate their anti-platelet and anti-cancer properties.

## 2. Results

### 2.1. Chemistry

**Scheme 1:**
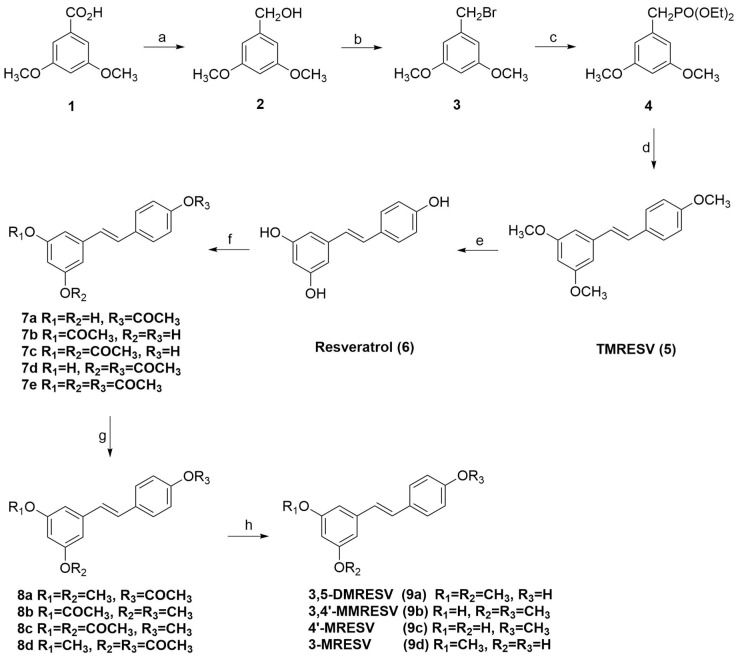
Reagents and conditions: (**a**) LiAlH_4_, THF dry, rt, 2 h; (**b**) CBr_4_, PPh_3_, CH_2_Cl_2_, rt, 2 h; (**c**) PO(Oet)_3_, Bu_4_NI, 120 °C; (**d**) 4-methoxybenzaldehyde, NaH, THF dry, rt; (**e**) BBr_3_, CH_2_Cl_2_ dry, rt, Ar; (**f**) Ac_2_O, Et_3_N, CH_2_Cl_2_, rt, 12 h; (**g**) (CH_3_)_2_SO_4_, Na_2_CO_3_, TEBAC, acetone, reflux; (**h**) Na_2_CO_3_, MeOH, H_2_O, rt.

The synthesis of methoxy-substituted resveratrol analogs is illustrated in Scheme 1. Resveratrol (**6**) was prepared using previously published procedures with slight modifications [30]. The process begins with the preparation of 3,5-dimethoxybenzylphosphonate **4**, which involves the reduction of benzoic acid **1** with LiAlH_4_ to afford benzyl alcohol **2**. Compound **2** was then reacted with CBr_4_ in the presence of PPh_3_, and the resulting bromide **3** is then reacted with PO(OEt)_3_ to yield the desired phosphonate **4**. A Wittig reaction of compound **4** with 4-methoxybenzaldehyde produces trimethoxyresveratrol **5**, which is further demethoxylated by treatment with BBr_3_, to obtain resveratrol **6**. Acetylation of resveratrol leads to a mixture of isomeric mono- di- or tri-acetylated resveratrol **7a–e**, which are separated by column chromatography and identified through 1D- and 2D-NMR experiments. The target methoxy compounds **9a–d** are obtained from the mono- and diacetylated resveratrol analogs (**7a,b** and **7c,d**, respectively) upon methylation and hydrolysis of the resulting methoxy analogs **8a–c**.

### 2.2. Biological Assays

Anti-platelet activity of methoxy resveratrol derivatives.

The anti-platelet activity of methoxy derivatives of resveratrol (Scheme 1) was evaluated by testing their ability to inhibit platelet aggregation in PRP from healthy volunteers. 

Three agonists of platelet aggregation were used, namely, PAF, ADP, and TRAP, and several concentrations of resveratrol and its methoxy derivatives were tested. The results are presented as IC_50_ values calculated either from the amplitude or from the AUC of the agonist and shown in Table 1.

Resveratrol was found to inhibit the aggregation of all agonists but not to the same extent (*p* < 0.000). It was observed that resveratrol inhibited platelet aggregation induced by PAF (*p* < 0.000) and TRAP (*p* = 0.001) more effectively than that induced by ADP. No significant difference was detected between its ability to inhibit PAF- and TRAP-induced platelet aggregation. Similar results were obtained when the IC_50_ values were calculated based on the AUC of aggregation instead of the amplitude, but lower IC_50_ values were revealed in all cases (*p* < 0.02). 

Methylation of resveratrol did not improve its ability to inhibit ADP-induced platelet aggregation. When methylation occurred in the 3-hydroxyl group (*p* < 0.000) or both in the 3- and 5-hydroxyl groups (*p* = 0.003) a significant decrease in inhibitory activity was observed compared to the initial compound. When methylation occurred in the 4′-hydroxyl group, i.e., the 4′ derivative (*p* = 0.13), the 3,4′ derivative (*p* = 1.0), and the 3,4′,5 derivative (*p* = 1.0), the IC_50_ values were higher but not significantly different from the initial compound. When the IC_50_ values were calculated based on the AUC of aggregation instead of amplitude, only methylation occurring in the 3′-hydroxyl group (*p* < 0.000) revealed a significant increase in IC_50_ value compared to resveratrol.

Regarding PAF as the agonist, methylation also worsened the ability of resveratrol to inhibit platelet aggregation (*p* < 0.000). Specifically, methylation in both aromatic rings (3- and 4′-hydroxyl groups) resulted in significantly higher IC_50_ values compared to the initial compound, as did trimethylation (3,4′-DMRESV *p* < 0.000, TMRESV *p* = 0.01). When methylation occurred only in one ring, no significant change was observed in IC_50_ values, although a slight increase was observed (3-MRESV *p* = 0.5, 4′-MRESV *p* = 0.4, 3,5-DMResv *p* = 0.3). Similar results were obtained when the IC_50_ values were calculated based on the AUC of aggregation instead of the amplitude (3,4-DMRESV *p* = 0.002, TMRESV *p* = 0.06, all the others *p* > 0.05).

**Figure 1:**
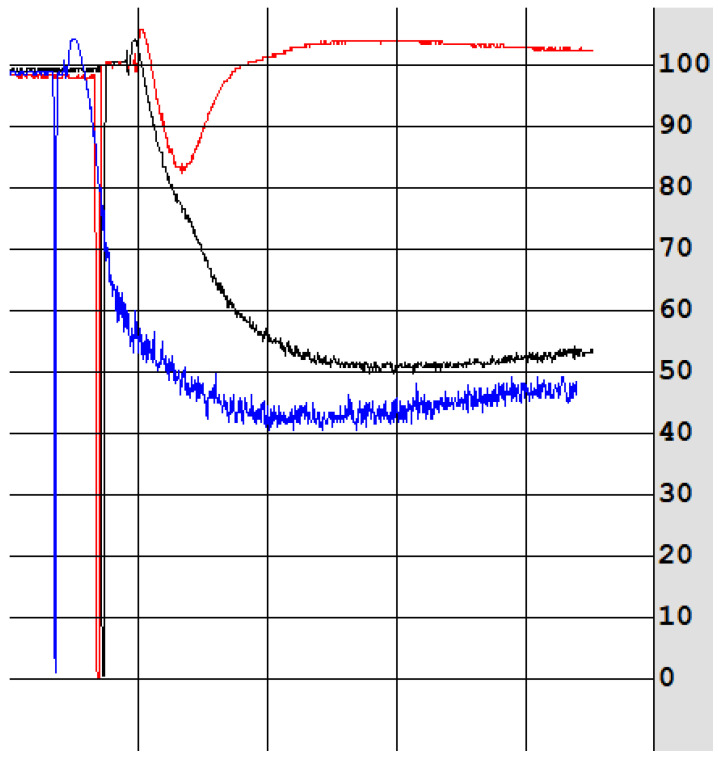
Characteristic inhibition of TRAP-induced platelet aggregation by 4′M-RESV. Blue line: DMSO (solvent) and TRAP; Black line: 6 6.7 μΜ 4′-MRESV and TRAP; Red line: 13.5 μΜ 4′-MRESV and TRAP.

In contrast, when TRAP was used as the agonist, methylation of resveratrol improved its ability to inhibit platelet aggregation. The monomethylated derivatives exhibited the best inhibitory ability, with almost one magnitude lower IC_50_ values, namely 3-MRESV (*p* < 0.000) and two 4′-MRESV (*p* < 0.000), respectively, compared to resveratrol. Dimethylation occurring in different rings resulted in a lower IC_50_ value (3,4′-DMRESV, *p* = 0.01), whereas when it occurred in the same ring, a similar IC_50_ was observed to the initial compound’s anti-platelet activity (3,5-DMRESV, *p* = 0.1). Figure 1 shows a characteristic inhibition of TRAP-induced platelet aggregation by 4′-MRESV. The trimethylation of resveratrol dramatically diminished its inhibitory activity, and the IC_50_ value could not be calculated. When the IC_50_ values were calculated based on AUC of aggregation instead of amplitude, similar results were obtained.

Anti-cancer effect of methoxy resveratrol derivatives

The anti-proliferative activity of the new compounds was evaluated in vitro against the PC-3 human prostate cancer cell line and the HCT116 colon cancer cell line. The results of the MTT dye reduction assay, expressed as 50% inhibitory concentrations (IC_50_) in µM, are depicted in Table 2. Each experiment was performed in triplicate and mean values ± SD are reported. Among the tested compounds, 3-MRESV is found to be more potent than resveratrol in androgen-independent prostate cancer cells (PC-3). In addition, 3,5-DMRESV and 3,4′-DMRESV exhibited significant anti-cancer properties to a lesser extent. Furthermore, 3,4′-DMRESV and TMRESV were found to be more potent than resveratrol in colon cancer cells (HCT116).

### 2.3. Molecular Calculations

To better understand the selectivity of the different methylated analogues of resveratrol, we run molecular docking simulations utilizing the induced-fit algorithm as implemented on Schrodinger Suite 2019. We selected the crystallographic structure of human protease-activated receptor 1 (PAR1) bound with the antagonist vorapaxar (PDB: 3VW7) as the starting structure. Vorapaxar, unlike ligands for other GPCRs, does not penetrate deeply into the transmembrane core. It is composed of two hydrophobic cores connected with a double bond, showing moderate similarity to resveratrol scaffold.

**Figure 2:**
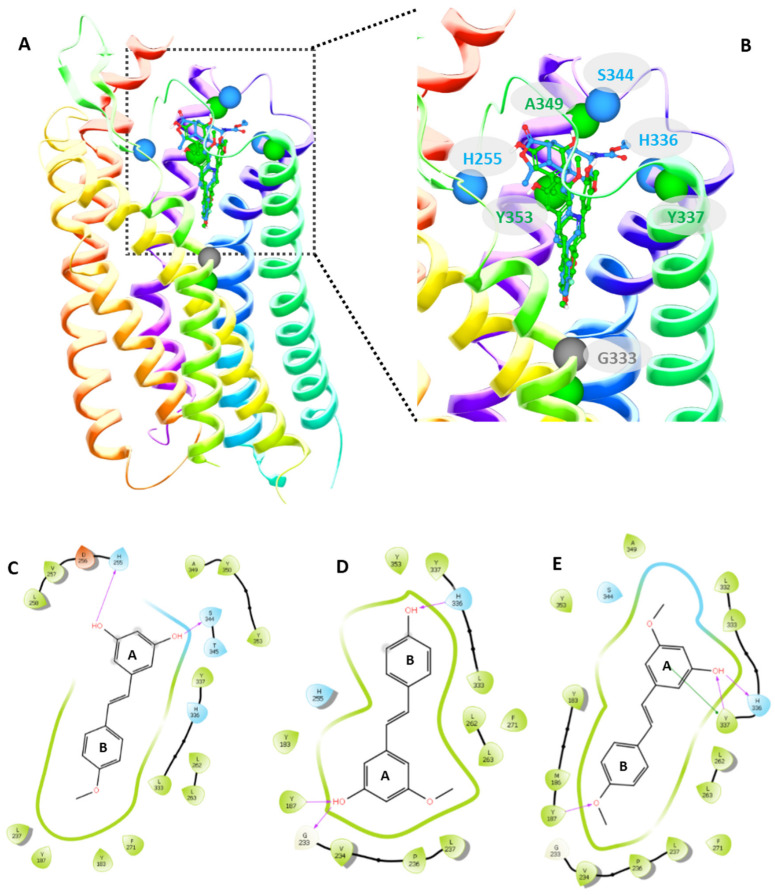
(**A**) Superposition of global minimum structures of most potent analogues with vorapaxar. Protein is represented with colored ribbons. Each of the 7 TM is uniquely colored. (**B**) Detail of the binding pocket. Residues interacting with each ligand are depicted. (**C**–**E**) 2D Interaction diagram for 4′-MRESV, 3-MRESV, and 3,4′-DMRESV, respectively.

All synthesized analogues were docked inside the binding cavity with average binding affinity (9–11 Kcal/mol) (Figure 2A,B). The 4′-methyl group of 4′-MRESV is oriented similarly to the fluorophenyl–pyridyl group of Vorapaxar, buried in a hydrophobic cavity (Figure 2C). The 2 hydroxyl groups are oriented next to the extracellular part of PAR1, forming hydrogen bonds with H255 and S344 (Figure 2C). 3-MRESV is oriented with the A phenyl group in the hydrophobic pocket. The 5 OH group forms hydrogen bonds with Y187 and the backbone of G233, while the 4′OH group forms hydrogen bond with H336 (Figure 2D). Moreover, 3,4′DMRESV is oriented deeper to the binding cavity because of the second O-Me group, which is placed next to Y353 and A349, while the phenolic OH group forms hydrogen bonds with H336 and Y337 (Figure 2E). 3,5-DMRESV and TMRESV are placed inside the binding cavity with the 3,5 dimethyl groups facing the extracellular aqueous environment, decreasing the overall binding affinity. In the case of 3,5-DMRESV, the OH group is placed inside the hydrophobic cavity, and although it is forming hydrogen bonds with Y187 and the backbone of G233, it is unfavorable to pass through such a hydrophobic channel and finally reach those residues. Resveratrol shows the lowest binding energy and has the 4′OH group placed inside the hydrophobic cavity which is unfavorable as was explained earlier.

## 3. Discussion

Resveratrol is a natural molecule that has attracted much scientific interest in recent decades [2]. Several biological activities have been attributed to resveratrol, including anti-oxidant, anti-inflammatory, and anti-cancer effects. However, its low availability is a primary concern about its in vivo activity. In this study, methoxy derivatives of resveratrol were synthesized to increase its hydrophobicity and were found to have similar, worse, or better anti-platelet activity than the initial molecule depending on the position of the substitution. The 4′ methoxy and 3 methoxy derivatives efficiently inhibit TRAP-induced platelet aggregation, especially in two orders and one order of magnitude, respectively, more potently than the initial compound.

Platelets are key cells in many physio-pathological mechanisms [14]. Several antagonists, such as thrombin, collagen, ADP, PAF, serotonin, epinephrine, and thromboxane A2 (TXA2), induce platelet activation and aggregation. Many reports indicate that resveratrol inhibits thrombin, collagen, PAF or ADP-induced platelet adhesion or aggregation [18,26]. In addition, resveratrol inhibited thromboxane B2-induced platelet aggregation by inhibiting protein kinase C activity [18]. The inhibitory capacity of resveratrol ranges between tens and hundreds of μM depending on the use of PRP or washed platelet preparations, the agonist used, and the platelets’ origin (human or rabbit). It should be noted that a lower concentration is needed when washed platelets are used to determine the anti-platelet effect. In the present study, human PRP was used instead of washed platelets because we believe that its matrix is more representative of the cells’ in vivo environment. The IC_50_ value of resveratrol against PAF and TRAP was similar, whereas against ADP, it was significantly higher, indicating a greater capacity of resveratrol to interfere with PAF and TRAP agonists instead of ADP.

Methylation has been shown to significantly affect platelet aggregation, depending on the number of methoxy groups and the specific methylated ring or position. Furthermore, the influence of methylation on platelet aggregation varies depending on the agonist used to induce it. ADP was the first low-molecular-weight compound identified as a platelet agonist [31] and stimulates primary platelet aggregation. Released by the storage granules, ADP is responsible for the secondary wave of aggregation and it is also involved in platelet stimulation by other agonists [32]. ADP-induced platelet aggregation is mediated by the activation of platelet P2Y1 and P2Y12 receptors [33]. However, resveratrol’s methylation did not improve the ability to inhibit ADP-induced platelet aggregation. In fact, when the substitution was carried out in the A ring, either in one or both hydroxyl groups, the anti-platelet activity of the initial compound worsened. In addition, the methylation in both the 3- and 5-hydroxy groups, in ring A has a lower impact on the second wave of platelet aggregation, as the IC_50_ value calculated based on AUC, instead of amplitude, was not significantly different from that of the initial compound. On the other hand, isorhapontigenin (3,4′,5-trihydroxy-3′-methoxystilbene), another derivative of resveratrol with an extra methylated hydroxyl group, has been reported to inhibit ADP-induced platelet aggregation more efficiently, which was also supported by molecular interactions of isorhapontigenin and resveratrol with P2Y12 receptor as predicted by in silico studies [34].

PAF, also known as acetyl-glyceryl-ether-phosphorylcholine, is an ether phospholipid that functions as a potent inducer of inflammation and platelet aggregation [12]. PAF exerts its effects through its specific receptor, PAFR, which is a G-protein coupled receptor with seven transmembrane domains. In our previous report, we demonstrated that acetylated derivatives inhibit PAF-induced platelet aggregation [26]. Specifically, mono- and tri-acetylation of resveratrol showed similar anti-platelet activity to resveratrol itself, while di-acetylation resulted in improved anti-platelet activity, indicating that the presence of two acetyl groups, but not three, is necessary for enhancing inhibitory activity. In the present study, methylation of resveratrol did not enhance its anti-platelet activity. Methylation of one ring (either A or B) did not show significant differences compared to the original compound. However, when methylation was performed on both rings (3,4′-DMRESV and TMRESV), resveratrol’s ability to inhibit PAF-induced aggregation was reduced. These findings suggest that the specific group used for substitution (acetoxy or methoxy) in resveratrol is more crucial than the increase in lipophilicity achieved by both substitutions.

Thrombin, a serine protease, is another enzyme that induces platelet aggregation and triggers the release of active substances, including ADP. This process requires the presence of calcium ions. Thrombin elicits signaling, partly through G-protein–linked protease-activated receptors (PARs), with human platelets expressing PAR1 and PAR4 [35]. PAR1 is considered to be the high-affinity thrombin receptor responsible for the initial platelet response, while PAR4 requires higher thrombin concentrations and participates in the late-phase response [36]. Consequently, PAR1 has attracted scientific interest as a novel anti-platelet target, but only one PAR1 antagonist, vorapaxar, is currently approved for clinical use [37]. In the present study, instead of thrombin, TRAP was used as agonist, a synthetic peptide that corresponds to the new N-terminal amino acid sequence of the “tethered ligand” generated after thrombin hydrolysis of the PAR1. The results revealed that, in some cases, methylation of resveratrol resulted in favorable alterations of its anti-platelet action. Specifically, dimethylation in the A ring (3,5-DMRESV, TMRESV) revealed compounds with similar or no anti-platelet activity against TRAP compared to resveratrol. In contrast, methyl substitution in the 3- or 4’-hydroxyl groups, alone or in combination, resulted in compounds with significantly enhanced anti-platelet activity. The dimethyl derivative (3,4′-DMRESV), the monomethyl derivative in the A ring (3-MRESV), and the monomethylated derivative in the B ring (4′-MRESV) exhibited IC_50_ values almost half, 3.5 times, and 25 times lower than resveratrol, respectively. To our knowledge, no similar data exists to compare our results. However, a recent study reported that the 3,5,2′,4′-tetramethoxystilbene selectively inhibits PAR4-mediated human platelet aggregation, ATP secretion, integrin αIIbβ3 activation, and signaling pathways [27].

The activation of platelets by all tested agonists involves the activation of phopholipase C, which hydrolyzes the membrane-associated phosphatidylinositol 4,5 bisphosphate and produces the second messenger inositol 1,4,5-irisphosphate (IP3), contributing to Ca^2+^ mobilization from internal stores [35]. Additionally, the production of membrane-associated diacylglycerol activates protein kinase C, which participates in the phosphorylation of specific substrates [35]. While the anti-platelet mechanisms of methoxy derivatives cannot be identified based on the design of this study, their distinct actions against different agonists suggest interference in specific pathways related to each agonist. In silico studies were conducted to further investigate the binding of resveratrol methoxy derivatives with PAR1, supporting the biological experiments and revealing that 4′-MRESV exhibits greater binding affinity toward the PAR1 receptor than resveratrol.

Among the new compounds, 3MRESV appears to be worth investigating in prostate cancer cells, along with 3,5-DMRESV and 3,4′-DMRESV, which have IC_50_ values ranging from 20.3–43 µM. Furthermore, 3,4′-DMRESV and TMRESV have demonstrated greater activity against colon cancer cells, with IC_50_ values ranging from 48.6–54.8 µM. These compounds could be considered for further optimization. Previous studies by Grau et al., have shown that the methoxy derivative of resveratrol (E)-[1-(3,4,5-Trimethoxyphenyl)-2-(2,4-dimethoxyphenyl)]ethene showed the strongest cytotoxicity against colon carcinoma (HT-29) and pancreatic carcinoma (MIA PaCa-2) cells lines with IC_50_ values of 12 ± 3 and 28 ± 4 μM, respectively, being six and five times more active than resveratrol in the respective cell lines, whereas (E)-1,3-Dimethoxy-5-(4-methoxystyryl)benzene also showed significant cytotoxicity 5- and 4-fold higher than resveratrol on HT-29 and MIA PaCa-2 cells [38]. In addition, Gosslau et al. demonstrated that the methoxy derivative of resveratrol 3,4,5,4′-tetramethoxy-*trans*-stilbene, inhibited the growth of LNCaP prostate cancer cells, HT-29 colon cancer cells, and HepG2 hepatoma cells with the IC_50_ values in the range of 1–5 μM [39]. Furthermore, 3,4,4′,5-tetramethoxy-stilbene demonstrated significant cytotoxic activity in clonogenic assays in the three cell lines investigated, i.e., 3 μM in the PC-3 cells, 0.4 μM in the LNCaP, and DU-145 prostate cancer cell lines instead of 16 μM, 5 μM, and 10 μM of resveratrol, respectively [40]. Furthermore, the para- and ortho- resveratrol derivatives appeared as the most active RSV derivatives in inhibiting cell viability in a panel of pancreatic cancer cell lines with distinct genetic profiles compared to resveratrol [41]. Finally, the resveratrol analogues 4,4′-(ethane-1,2-diyl) bis(2-methoxyphenol) and 4-(3-hydroxy-4-methoxyphenethyl)-2-methoxyphenol had very similar structures but exerted different cytotoxicity in non-small lung cancer cells. The IC50 values were 108.6 ± 10.82 and more than 200 µM in the A549 cell line and 103.5 ± 6.08 and more than 200 µM in H23 cells, respectively, while resveratrol itself exhibited more than 200 µM in both cell lines [42].

Finally, aspirin (acetylsalicylic acid) has emerged as a promising agent for cancer prevention. However, there are suggestions that anti-platelet treatment may promote the development of solid tumors. Given the complex and bidirectional interaction between tumor cells and platelets in the blood and tumor microenvironment, further investigation is needed to understand the potential of anti-platelet agents in both cancer prevention and treatment [28,29].

## 4. Materials and Methods

### 4.1. General Information

All commercially available chemicals were purchased from Alfa Aesar. ^1^H NMR, ^13^C NMR, and 2D spectra (HMBC, HMQC, and COSY) were recorded on a Bruker Avance III 600 and 400 spectrometer (Bruker GmbH, Bremen, Germany) using dimethyl sulfoxide (DMSO-*d_6_*), methanol (CD_3_OD), acetone (CD_3_COCD_3_), and chloroform (CDCl_3_) as deuterated solvents and were referenced to TMS (*δ* scale). Flash chromatography was performed on Merck silica gel 60 (0.040–0.063 mm). Analytical thin layer chromatography (TLC) was carried out on pre-coated silica gel F-254 plates (0.25 mm, Merck KgaA, Darmstadt, Germany). 

### 4.2. Synthesis of Methoxyresveratrol Analogs

3,5-Dimethoxybenzyl alcohol (**2**)

To a suspension of LiAlH_4_ (1.52 g, 40 mmol) in dry THF (50 mL), at 0 °C, under argon, was added dropwise a solution of acid **1** (1.82 g, 10 mmol) in dry THF (10 mL) and the resulting solution was stirred at rt for 2 h. After completion of the reaction, the mixture was cooled at 0 °C and carefully triturated with 10% aqueous NaOH solution and the resulting precipitate was filtrated through a Celite pad and washed with a small amount of THF. The filtrate was diluted with water, extracted with EtOAc (3 × 100 mL), dried over anhydrous Na_2_SO_4_, and concentrated to dryness, to afford the title compound **2** (1.2 g, 72%) which was used for the next step without any further purification [30]. ^1^H NMR (600 MHz, CDCl_3_) δ (ppm) 6.51 (d, *J* = 2.1 Hz, 2H), 6.32 (t, *J* = 2.1 Hz, 1H), 4.50 (s, 2H), 3.73 (s, 6H). ^13^C NMR (151 MHz, CDCl_3_) δ (ppm) 161.2, 143.7, 104.6, 99.7, 65.2, 55.4.

3,5-Dimethoxybenzyl bromide (**3**)

To a solution of alcohol **2** (0.84 g, 5 mmol) in dry CH_2_Cl_2_ (100 mL), at 0 °C, under argon, was added PPh_3_ (5.24 g, 20 mmol, 1.2 eq.), and CBr_4_ (4.98 g, 15 mmol) and the resulting solution was stirred at rt for 2 h. After completion of the reaction, the volatiles were vacuum evaporated and the crude product was purified by column chromatography (silica gel), using a mixture of cyclohexane—EtOAc 5—1 as the eluent, to afford 728 mg (63%) of the title compound **3** [30]. ^1^H NMR (600 MHz, CDCl_3_) δ (ppm) 6.53 (s, *J* = 2.1 Hz, 2H), 6.39 (s, *J* = 2.1 Hz, 1H), 4.38 (s, 2H), 3.81 (s, 6H). ^13^C NMR (151 MHz, CDCl_3_) δ (ppm) 161.1, 139.9, 107.2, 100.3, 55.6, 33.4.

Diethyl (3,5-dimethoxybenzylphosphonate) (**4**)

A mixture of 3,5-dimethoxybenzyl bromide (1.16 g, 5 mmol, **3**) and Bu_4_NI (184 mg, 0.5 mmol) in PO(OEt)_3_ (10 mL) was stirred at 120 °C for 12 h, under argon. After completion of the reaction, the volatiles were vacuum evaporated to afford the title compound **4**, which was used for the next step without any further purification [30]. ^1^H NMR (600 MHz, CDCl_3_) δ (ppm) 6.50 (d, *J* = 2.1 Hz, 2H), 6.36 (d, *J* = 2.1 Hz, 2H), 4.04 (m, 4H), 3.80 (s, 6H), 3.07–3.09 (s, 2H), 1.26 (t, *J* = 7.0 Hz, 6H).

(E)-3,4′,5-trimethoxystilbene or trimethoxyresveratrol (**5**, **TMRESV**)

To a solution of diethyl 3,5-dimethoxybenzylphosphonate (1.15 g, 4 mmol, **4**) and *p*-anisaldehyde (558 mg, 4.1 mmol) in dry THF (10 mL), at 0 °C, under argon, was added portion-wise NaH (180 mg, 4.5 mmol, 60% in mineral oil) and the resulting mixture was stirred at rt for 16 h. After completion of the reaction, the mixture was cooled at 0 °C, carefully triturated with methanol, and evaporated to dryness. The residue was dissolved in CH_2_Cl_2_, washed with water, brine, dried over anhydrous Na_2_SO_4_, and evaporated to dryness [30]. ^1^H NMR (600 MHz, CDCl_3_) δ (ppm) 7.39 (d, *J* = 8.6 Hz, 2H), 7.00 (d, *J* = 16.3 Hz, 1H), 6.84–6.92 (m, 3H), 6.56 (d, *J* = 2.1 Hz, 2H), 6.42 (t, *J* = 2.2 Hz, 1H), 3.82 (s, 9H). ^13^C NMR (151 MHz, CDCl_3_) δ (ppm) 160.2, 159.4, 140.2, 130.0, 129.2, 128.6, 127.5, 126.0, 114.1, 103.2, 100.3, 55.6.

(E)-5-(4-Hydroxystyryl)benzene-1,3-diol or resveratrol (**6**)

To a solution of **5** (2.16 g, 8 mmol) in dry CH_2_Cl_2_ (45 mL) at −78 °C, under argon, was added dropwise a solution of BBr_3_ (25 mL, 25 mmol, 1 M solution in CH_2_Cl_2_) and the resulting mixture was stirred at rt for 4 h. After completion of the reaction, the mixture was cooled at 0 °C and carefully poured into ice. The organic phase was separated, dried over Na_2_SO_4_, and concentrated under reduced pressure. The aqueous phase was filtered, the filtrate was washed with water and air-dried. The crude product (from aqueous and organic phase) was purified by column chromatography (silica gel) using a mixture of cyclohexane—EtOAc 1—1 as the eluent, to obtain pure compound **6** (1.44 g, 79%). ^1^H NMR (600 MHz, CD_3_COCD_3_) δ (ppm) 8.40 (s, 1H, OH), 8.23 (s, 2H, 2xOH), 7.40 (d, *J* = 8.4 Hz, 2H), 7.00 (d, *J* = 16.4 Hz, 1H), 6.89 (d, *J* = 16.4 Hz, 1H), 6.79 (d, *J* = 8.4 Hz, 2H), 6.57 (d, *J* = 2.0 Hz, 2H), 6.28 (t, *J* = 2.0 Hz, 1H). ^13^C NMR (151 MHz, DMSO-*d6*) δ (ppm) 159.2, 157.6, 139.3, 128.1, 127.9, 125.7, 115.6, 104.4, 101.9.

Synthesis of acetylated resveratrol compounds **7a–e**

A suspension of resveratrol (950 mg, 4.17 mmol, **6**), acetic anhydride (553 μL, 5.84 mmol) and triethylamine (1.8 mL, 11.68 mmol) in anhydrous CH_2_Cl_2_ (100 mL), was stirred at room temperature for 12 h. The reaction mixture was then vacuum evaporated, extracted with CH_2_Cl_2_ and water, the organic layer was dried over Na_2_SO_4_ and evaporated to dryness. The residue was purified by column chromatography (silica gel) using a mixture of CH_2_Cl_2_—EtOAc 100—4 as the eluent, to afford the title compounds **7a–e** [43]. 

Spectral data for (E)-4-(3,5-dihydroxystyryl)phenyl acetate (**7a**): 1H NMR (600 MHz, CD_3_OD) δ (ppm) 7.55 (d, *J* = 8.5 Hz, 2H), 7.09 (d, *J* = 8.5 Hz, 2H), 7.05 (d, *J* = 16.2 Hz, 1H), 6.99 (d, *J* = 16.2 Hz, 1H), 6.52 (d, *J* = 2 Hz, 2H), 6.23 (t, *J* = 2 Hz, 1H), 2.29 (s, 3H). ^13^C NMR (151 MHz, CD_3_OD) δ (ppm) 169.7, 158.3, 150.1, 139.2, 135.1, 128.8, 126.9, 126.9, 121.5, 104.7, 101.8, 19.4.

Spectral data for (E)-3-hydroxy-5-(4-hydroxystyryl)phenyl acetate (**7b**): ^1^H NMR (600 MHz, CD_3_OD) δ (ppm) 7.40 (d, *J* = 8.5 Hz, 2H), 7.05 (d, *J* = 16.3 Hz, 1H), 6.89 (d, *J* = 16.3 Hz, 1H), 6.85 (t, *J* = 2.1 Hz, 1H), 6.80 (d, *J* = 8.5 Hz, 2H), 6.75 (t, *J* = 2.1 Hz, 1H), 6.42 (t, *J* = 2.1 Hz, 1H), 2.29 (s, 3H). ^13^C NMR (151 MHz, CD_3_OD) δ (ppm) 170.0, 158.1, 157.2, 151.9, 140.1, 129.9, 129.1, 128.7, 127.5, 124.5, 115.0, 110.1, 109.9, 107.2, 19.5.

Spectral data for (E)-3-acetoxy-5-(4-hydroxystyryl)phenyl acetate (**7c**):^1^H NMR (600 MHz, CDCl_3_) δ (ppm) 7.39 (d, *J* = 8.4 Hz, 2H), 7.11 (d, *J* = 2.0 Hz, 2-H), 7.03 (d, *J* = 16.2 Hz, 1H), 6.89 (d, *J* = 16.2 Hz, 1H), 6.84(d, *J* = 8.4 Hz, 2H), 6.82 (t, *J* = 2.1 Hz, 1H), 2.34 (s, 6H). ^13^C NMR (151 MHz, CDCl_3_) δ (ppm) 169.1, 155.8, 151.3, 140.1, 130.2, 129.6, 128.2, 124.8, 116.7, 115.7, 113.9, 21.2.

Spectral data for (E)-4-(3-hydroxy-5-acetoxystyryl)phenyl acetate(**7d**):^1^H NMR (600 MHz, CDCl_3_) δ (ppm) 7.49 (d, *J* = 8.4 Hz, 2H), 7.11 (d, *J* = 8.4 Hz, 2H), 7.03 (d, *J* = 16.3 Hz, 1H), 6.93 (d, *J* = 16.3 Hz, 1H), 6.84 (br s, 1H), 6.81 (br s, 1H), 6.53 (br s, 1H), 5.28 (br s, 1H), 2.34 (s, 3H), 2.34 (s, 3H). ^13^C NMR (151 MHz, CDCl_3_) δ (ppm) 169.6, 169.5, 156.6, 151.8, 150.3, 139.7, 134.7, 129.0, 127.8, 127.6, 121.9, 112.0, 111.1, 108.5, 21.2.

Spectral data for (E)-4-(3,5-diacetoxystyryl)phenyl acetate (**7e**): ^1^H NMR (600 MHz, CDCl_3_) δ (ppm) 7.51 (d, *J* = 8.5 Hz, 2H), 7.15 (d, *J* = 1.9 Hz, 2H), 7.12 (d, *J* = 8.5 Hz, 2H), 7.09 (d, *J* = 16.3 Hz, 1H), 7.00 (d, *J* = 16.3 Hz, 1H), 6.86 (t, *J* = 2.0 Hz, 1H), 2.33 (s, 9H). ^13^C NMR (151 MHz, CDCl_3_) δ (ppm) 169.3, 168.9, 151.3, 150.4, 139.5, 134.4, 129.6, 127.6, 127.1, 121.8, 116.8, 114.3, 21.0.

(E)-4-(3,5-dimethoxystyryl)phenyl acetate (**8a**)

To a solution of **7a** (78 mg, 0.29 mmol) in dry acetone (20 mL) was added Na_2_CO_3_ (60 mg, 0.58 mmol), benzyltriethylammonium chloride (15 mg) and dimethyl sulfate (55 μL, 0.58 mmol) and the resulting mixture was stirred under reflux for 48 h. After completion of the reaction, the mixture was evaporated to dryness and the residue was dissolved in CH_2_Cl_2_, washed water, dried over Na_2_SO_4_, and concentrated to dryness. The crude product was purified by column chromatography (silica gel) using a mixture of cyclohexane—EtOAc 1—1 as the eluent, to obtain pure compound **8a** (54 mg, 63%) [44]. ^1^H NMR (400 MHz, CD_3_OD) δ (ppm) 7.48 (d, *J* = 8.7 Hz, 2H), 7.11 (d, *J* = 16.4 Hz, 1H), 7.00–6.93 (m, 2H), 6.91 (d, *J* = 8.7 Hz, 2H), 6.87 (s, 1H), 6.57 (t, *J* = 2.0 Hz, 1H), 3.82 (s, 3H), 3.81 (s, 3H), 2.29 (s, 3H).

(E)-3-methoxy-5-(4-methoxystyryl)phenyl acetate (**8b**)

This compound was prepared by an analogous procedure as described for the preparation of **8a** using compound **7b**. Yield: 66% [45]. ^1^H NMR (400 MHz, CDCl_3_) δ (ppm) 7.42 (d, *J* = 8.8 Hz, 2H), 7.03 (d, *J* = 16.2 Hz, 1H), 6.90–6.87 (m, 4H), 6.84 (d, *J* = 2.1 Hz, 1H), 6.55 (t, *J* = 2.0 Hz, 1H), 3.82 (s, 3H), 3.81 (s, 3H), 2.29 (s, 3H).

(E)-5-(4-methoxystyryl)-1,3-phenylene diacetate (**8c**)

This compound was prepared by an analogous procedure as described for the preparation of **8a** using compound **7c**. Yield: 60% [46]. ^1^H NMR (600 MHz, CD_3_COCD_3_) δ (ppm) 7.62 (d, *J* = 8.6 Hz, 3H), 7.28 (d, *J* = 16.4 Hz, 2H), 7.18 (d, *J* = 16.4 Hz, 2H), 7.13 (d, *J* = 8.6 Hz, 3H), 7.04 (d, *J* = 1.3 Hz, 2H), 6.95 (s, 1H), 6.63 (t, *J* = 2.0 Hz, 1H), 3.83 (s, 5H), 2.26 (s, 9H).

(E)-4-(3-acetoxy-5-methoxystyryl)phenyl acetate (**8d**)

This compound was prepared by an analogous procedure as described for the preparation of **8a** using compound **7c**. Yield: 70% [47]. ^1^H NMR (600 MHz, CD_3_COCD_3_) δ (ppm) 7.62 (d, *J* = 8.6 Hz, 3H), 7.28 (d, *J* = 16.4 Hz, 2H), 7.18 (d, *J* = 16.4 Hz, 2H), 7.13 (d, *J* = 8.6 Hz, 3H), 7.04 (d, *J* = 1.3 Hz, 2H), 6.95 (s, 1H), 6.63 (t, *J* = 2.0 Hz, 1H), 3.83 (s, 5H), 2.26 (s, 9H).

(E)-4-(3,5-dimethoxystyryl)phenol or 3,5-dimethoxyresveratrol (**9a**,** 3,5-DMRESV**)

To a solution of the ester **8a** (51 mg, 0.17 mmol) in a methanol/water solution (5 mL/5 mL) was added Na_2_CO_3_ (60 mg, 0.58 mmol) and the resulting mixture was stirred at rt for 2 h. After completion of the reaction, the mixture was evaporated to dryness and the residue was dissolved in CH_2_Cl_2_, washed with water, dried over anhydrous Na_2_SO_4_, and concentrated to dryness. The crude product was purified by column chromatography (silica gel) using a mixture of cyclohexane—EtOAc 4—1 as the eluent, to obtain pure compound **9a** (23 mg, 54%) [48]. ^1^H NMR (600 MHz, CD_3_COCD_3_) δ (ppm) 7.39 (d, *J* = 8.5 Hz, 2H), 7.04 (d, *J* = 16.5 Hz, 1H), 6.91 (d, *J* = 16.5 Hz, 1H), 6.85 (d, *J* = 8.5 Hz, 2H), 6.66 (d, *J* = 2.0 Hz, 2H), 6.37 (t, *J* = 2.0 Hz, 1H), 3.80 (s, 6H). ^13^C NMR (151 MHz, CD_3_COCD_3_) δ (ppm) 160.7, 156.4, 140.5, 129.8, 129.1, 127.5, 125.9, 115.8, 104.6, 99.4, 54.9.

(E)-3-methoxy-5-(4-methoxystyryl)phenol or 3,4′-dimethoxyresveratrol (**9b**,** 3,4′-DMRESV**)

This compound was prepared by an analogous procedure as described for the preparation of **9a** using compound **8b**. Yield: 66% [49]. ^1^H NMR (600 MHz, CDCl_3_) δ (ppm) 7.45 (d, *J* = 8.4 Hz, 2H), 7.00(d, *J* = 16.0 Hz, 1H), 6.86–6.91 (m, 3H), 6.64 (d, *J* = 1.6 Hz, 1H,), 6.61 (t, *J* = 1.6 Hz, 1H), 6.35 (t, *J* = 1.6 Hz, 1H), 3.85 (s, 3H), 3.83 (s, 3H). ^13^C NMR (151 MHz, CDCl_3_) **δ** (ppm) 160.5, 158.5, 156.8, 139.5, 129.4, 128.7, 128.3, 125.7, 114.2, 105.8, 103.8, 100.5, 55.4, 55.3.

(E)-5-(4-methoxystyryl)benzene-1,3-diol or 4′-methoxyresveratrol (**9c**, **4′-MRESV**)

This compound was prepared by an analogous procedure as described for the preparation of **9a** using compound **8c**. Yield: 60% [49]. ^1^H NMR (600 MHz, CD_3_COCD_3_) δ (ppm) 7.47 (d, *J* = 8.5 Hz, 2H), 7.08 (d, *J* = 16.3 Hz, 2H), 6.93–6.82 (m, 3H), 6.65 (d, *J* = 1.9 Hz, 2H), 6.33 (t, *J* = 2.1 Hz, 1H), 3.79 (s, 3H). ^13^C NMR (151 MHz, CD_3_COCD_3_) δ (ppm) 160.1, 158.1, 141.23, 130.1, 129.2, 128.1, 127.0, 114.2, 105.8, 102.1, 55.8.

(E)-3-(4-hydroxystyryl)-5-methoxyphenol or 3-methoxyresveratrol (**9d**,** 3-MRESV**)

This compound was prepared by an analogous procedure as described for the preparation of **9a** using compound **8d**. Yield: 69% [49]. ^1^H NMR (600 MHz, CD_3_COCD_3_) δ (ppm) 7.47 (d, *J* = 8.5 Hz, 2H), 7.08 (d, *J* = 16.3 Hz, 2H), 6.93–6.82 (m, 3H), 6.65 (d, *J* = 1.9 Hz, 2H), 6.33 (t, *J* = 2.1 Hz, 1H), 3.79 (s, 3H). ^13^C NMR (151 MHz, CD_3_COCD_3_) δ (ppm)160.1, 158.1, 141.23, 130.1, 129.2, 128.1, 127.0, 114.2, 105.8, 102.1, 55.8.

### 4.3. Blood Sample Collection 

The study was conducted at the Metabolic Unit of the Department of Nutrition and Dietetics, Harokopio University, according to the guidelines laid down in the Declaration of Helsinki. Volunteers gave informed consent and the University Ethics Committee approved the experimental protocol (number: 57/15 September 2017). Informed consent was obtained from all subjects involved in the study. Healthy volunteers who had not taken aspirin or non-steroidal anti-inflammatory drugs for at least 2 weeks were used. Twelve hour fasting venous blood was collected from the brachial vein of the volunteers using citrate acid as anticoagulant. Blood was centrifuged at 170× *g* for 15 min in order to collect the supernatant platelet-rich plasma (PRP). Platelet-poor plasma (PPP) was obtained by recentrifugation of the pellet at 1500× *g* for 10 min.

### 4.4. Platelet Aggregation in Plasma-Rich Platelets (PRP)

The effect of phenolic compounds on platelet aggregation against PAF, ADP, and TRAP (Thrombin analogue) was determined on plasma-rich platelets (PRP) from healthy volunteers. The molecules were dissolved in DMSO (1.6% maximum DMSO final concentration). Platelet count of PRP was adjusted to 300.000/mL with PPP. Samples were incubated at 37 °C, with a stirring rate of 1.000 rpm. A Chronolog Aggregometer (Model 440VS) was used for determining aggregation responses based on the light transmittance method against various concentrations of PAF, ADP, and thrombin receptor-activating peptide (TRAP), a PAR-1 (Protease-Activated Receptor) selective activating peptide. Optical aggregation results were expressed as a percentage of aggregation at a given time interval from agonist addition; aggregation was defined as the difference between the 0% (PRP) baseline and the 100% (PPP) baseline. The aggregation was induced by final concentration of PAF ranging between 1–4 μM (dissolved in bovine serum albumin 2.5 mg/mL), of ADP ranging between 50–100 μM (dissolved in saline) and of TRAP ranging between 4–7 μM (dissolved in saline) with respect to each volunteer. In addition, 0% inhibition was considered as platelet aggregation without the addition of the examined compound. The plot of percentage inhibition (ranging from 20% to 80%) vs. different concentrations of the examined compound is linear and it was used to calculate the concentration of the sample that induced 50% inhibition against each agonist. This value was defined as the IC_50_, namely, inhibitory concentration producing 50% inhibition. 

### 4.5. Cell Lines

Human HCT116 colon cancer cell lines and PC-3 prostate cancer cell lines were obtained from the American Type Cell Culture (ATCC, Bethesda, MD, USA). Both cell lines were grown in 75 cm^2^ culture flasks at 37 °C in 5% CO_2_ using Roswell Park Memorial Institute 1640 medium (RPMI 1640, Gibco, Waltham, MA, USA) and Dulbecco’s modified Eagle’s medium F/12 (DMEM/F-12, Gibco) respectively, containing 10% fetal bovine serum (FBS, Gibco).

### 4.6. MTT Assay

To test the inhibitory activities of compounds using a cell-based protocol, MTT assay was performed for cell viability. HCT116 cells were plated at a density of 1500 per well, while PC-3 cells were plated at a density of 750 per well in a 96-well plate. After 24 h, cells were treated with the compounds in a dose-dependent manner for 72 h and 96 h. MTT [3-(4,5-dimethylthiazol-2-yl)-2,5-diphenyltetrazolium bromide] (Sigma M-5655) was added at a final concentration of 0.5 mg/mL directly to each well for 4 h at 37 °C. The medium was aspirated and the blue MTT formazan precipitate was dissolved in dimethyl sulfoxide (DMSO). Absorbance was determined in a Powerwave microplate spectrophotometer (Biotek Instruments, Inc., Winooski, VT, USA) at 540 nm. Viable cell numbers were determined by tetrazolium conversion to its formazan dye. Each experiment was performed in triplicate [50].

### 4.7. Molecular Calculations

Protein Preparation

The protein (PDB entry 3VW7) was prepared for the docking calculations using the Protein Preparation Workflow (Schrödinger Suite 2021 Protein Preparation Wizard) implemented in Schrödinger suite and accessible from within the Maestro program (Maestro, version 12.8, Schrödinger, LLC, New York, NY, USA, 2021). Briefly, the hydrogen atoms were added and the orientation of the hydroxyl groups, Asn, Gln, and the protonation state of His were optimized to maximize hydrogen bonding. Finally, the ligand–protein complex was refined with a restrained minimization performed by Impref utility, which is based on the Impact molecular mechanics engine (Impact version 9.1, Schrödinger, LLC, New York, NY, USA, 2021) and the OPLS_2005 force field, setting a max rmsd of 0.30. Ligand preparation for docking was performed with the LigPrep (Schrödinger, LLC, New York, NY, USA, 2021) application which consists of a series of steps that perform conversions, apply corrections to the structure, generate ionization states and tautomers, and optimize the geometries.

Ligand Preparation

All ligands were designed using Maestro software (Maestro, version 12.8, Schrödinger, LLC, New York, NY, USA, 2021). Furthermore, LigPrep (Schrödinger, LLC, New York, NY, USA, 2021) was used to generate tautomeric, stereochemical, and ionization variations for all ligands. Finally, partial charges were predicted from the force field OPLS2005.

Docking Simulations

The induced-fit docking algorithm was utilized for molecular docking as implemented on Schrödinger Suite 2021. For calculating the grid box size, the center of the grid box was taken to be the center of the ligand in the crystal structure, and the length of the grid box for the receptor was twice the distance from the ligand center to its farthest ligand atom plus 10 Å in each dimension. The scoring calculations were performed using standard precision (SP).

### 4.8. Statistical Analysis 

Statistical analysis was completed by using SPSS 18 (SPSS Inc., Chicago, Il, USA) software. Kolmogorov–Smirnov criterion was used for testing normality. All variables were normally distributed and presented as mean values ± standard deviation. Significance level for *p*-values is 5%.

## 5. Conclusions

In conclusion, the findings of this study support the notion that certain methoxy derivatives of resveratrol may have comparable or even better anti-platelet effects compared to the original compound. The most potent derivative observed was the 4′-methoxy derivative, which exhibited approximately 2.5 orders of magnitude greater anti-platelet activity against TRAP-induced platelet aggregation, suggesting its potential as an anti-platelet agent. Using in silico docking simulations, we have discovered that all compounds exhibit a binding mode comparable to vorapaxar. Furthermore, our investigation has allowed us to elucidate the correlation between binding modes and the potency of the 4′-methoxy derivative. It is also noteworthy that methoxy derivatives with similar activity to resveratrol could be potential anti-platelet agents as they may undergo in vivo bio-transformation, leading to improved bioavailability compared to resveratrol. These encouraging preliminary findings will provide valuable insights for our ongoing rational drug design campaign targeting human protease-activated receptor 1 (PAR1).

## Figures and Tables

**Table 1:** Anti-platelet effects of resveratrol and its methoxy derivatives against ADP, PAF, and TRAP as agonists. The results are expressed as IC_50_ values ± standard deviation of at least three independent experiments.

Compounds		IC_50_ ADP (mΜ)	IC_50_ PAF (mΜ)	IC_50_ TRAP (mΜ)
Resveratrol	Amplitude	0.476 ± 0.18 ^a^	0.165 ± 0.05 ^b^	0.224 ± 0.09 ^b^
	AUC	0.358 ± 0.20 ^a^	0.083 ± 0.05 ^b^	0.113 ± 0.04 ^b^
3-MRESV	Amplitude	>1 *	0.334 ± 0.05	0.063 ± 0.02
	AUC	>1 *	0.090 ± 0.06	0.039 ± 0.01
4′-MRESV	Amplitude	0.899 ± 0.33 ^a^	0.336 ± 0.19	0.009 ± 0.005
	AUC	0.352 ± 0.07	0.178 ± 0.09	0.004 + 0.002
3,4′-DMRESV	Amplitude	0.663 ± 0.13 ^b^	0.984 ± 0.20 *	0.110 ± 0.02
	AUC	0.512 ± 0.16	0.492 ± 0.29 *	0.038 ± 0.012
3,5-DMRESV	Amplitude	>1 *	0.351 ± 0.11	0.138 ± 0.05
	AUC	0.747 ± 0.15 *	0.174 ± 0.06	0.106 ± 0.04
TMRESV	Amplitude	0.721 ± 0.33 ^a^	0.528 ± 0.02 *	>1 *
	AUC	0.747 ± 0.15	0.368 ± 0.04 *	>1 *

Different letters indicate significant differences among the three agonists for the same phenolic compound. * Indicate significant differences among the derivatives and the resveratrol.

**Table 2:** Anti-proliferative activity of resveratrol and its methoxy derivatives. The results are expressed as IC_50_ values ± standard deviation of at least three independent experiments.

Compounds	PC-3	HCT116
Resveratrol	27.8 ± 3.2	76 ± 5.7
3-MRESV	20.3 ± 2.0 *	70.2 ± 5.4
4′-MRESV	>100	>100
3,4′-DMRESV	43.0 ± 0.6 *	54.8 ± 4.2 *
3,5-DMRESV	31.2 ± 1.9	84.9 ± 10.2
TMRESV	83.2 ± 5.9 *	48.6 ± 2.4 *

* Indicate significant differences among the derivatives and the resveratrol.

## Data Availability

The data presented in this study are available on request from the corresponding author.

## Sample Availability

Samples of the compounds are available from the authors.
